# Genomic Signatures of Strain Selection and Enhancement in *Bacillus atrophaeus* var. *globigii*, a Historical Biowarfare Simulant

**DOI:** 10.1371/journal.pone.0017836

**Published:** 2011-03-25

**Authors:** Henry S. Gibbons, Stacey M. Broomall, Lauren A. McNew, Hajnalka Daligault, Carol Chapman, David Bruce, Mark Karavis, Michael Krepps, Paul A. McGregor, Charles Hong, Kyong H. Park, Arya Akmal, Andrew Feldman, Jeffrey S. Lin, Wenling E. Chang, Brandon W. Higgs, Plamen Demirev, John Lindquist, Alvin Liem, Ed Fochler, Timothy D. Read, Roxanne Tapia, Shannon Johnson, Kimberly A. Bishop-Lilly, Chris Detter, Cliff Han, Shanmuga Sozhamannan, C. Nicole Rosenzweig, Evan W. Skowronski

**Affiliations:** 1 BioSciences Division, Edgewood Chemical Biological Center, Aberdeen Proving Ground, Maryland, United States of America; 2 Battelle Memorial Institute, Aberdeen Proving Ground, Maryland, United States of America; 3 Science Applications International Corporation, Aberdeen Proving Ground, Maryland, United States of America; 4 Johns Hopkins University Applied Physics Laboratory, Laurel, Maryland, United States of America; 5 Department of Bacteriology, University of Wisconsin, Madison, Wisconsin, United States of America; 6 OptiMetrics Inc, Abingdon, Maryland, United States of America; 7 Naval Medical Research Center, Biological Defense Research Directorate, Silver Spring, Maryland, United States of America; 8 Defense Threat Reduction Agency, Fort Belvoir, Virginia, United States of America; 9 The MITRE Corporation, McLean, Virginia, United States of America; 10 Department of Energy Joint Genome Institute, Los Alamos National Laboratories, Los Alamos, New Mexico, United States of America; 11 Excet Inc., Aberdeen Proving Ground, Maryland, United States of America; J. Craig Venter Institute, United States of America

## Abstract

**Background:**

Despite the decades-long use of *Bacillus atrophaeus* var. *globigii *(BG) as a simulant for biological warfare (BW) agents, knowledge of its genome composition is limited. Furthermore, the ability to differentiate signatures of deliberate adaptation and selection from natural variation is lacking for most bacterial agents. We characterized a lineage of BGwith a long history of use as a simulant for BW operations, focusing on classical bacteriological markers, metabolic profiling and whole-genome shotgun sequencing (WGS).

**Results:**

Archival strains and two “present day” type strains were compared to simulant strains on different laboratory media. Several of the samples produced multiple colony morphotypes that differed from that of an archival isolate. To trace the microevolutionary history of these isolates, we obtained WGS data for several archival and present-day strains and morphotypes. *Bacillus*-wide phylogenetic analysis identified *B. subtilis* as the nearest neighbor to *B. atrophaeus*. The genome of *B. atrophaeus* is, on average, 86% identical to *B. subtilis* on the nucleotide level. WGS of variants revealed that several strains were mixed but highly related populations and uncovered a progressive accumulation of mutations among the “military” isolates. Metabolic profiling and microscopic examination of bacterial cultures revealed enhanced growth of “military” isolates on lactate-containing media, and showed that the “military” strains exhibited a hypersporulating phenotype.

**Conclusions:**

Our analysis revealed the genomic and phenotypic signatures of strain adaptation and deliberate selection for traits that were desirable in a simulant organism. Together, these results demonstrate the power of whole-genome and modern systems-level approaches to characterize microbial lineages to develop and validate forensic markers for strain discrimination and reveal signatures of deliberate adaptation.

## Introduction

*Bacillus atrophaeus* is a soil-dwelling, non-pathogenic, aerobic spore-forming bacillus related to *B. subtilis*. For more than six decades, this organism has played an integral role in the biodefense community as a simulant for biological warfare and bioterrorism events (BW) and is commonly referred to by its military two-letter designation “BG” [1], [2]. *B. atrophaeus* has served in studies of agent dispersal [3], decontamination simulations [4], [5] and large-scale process development [6]. In addition to its historical use as a BW simulant, it is currently in widespread commercial use as a surrogate for spore-forming bacteria [5], [7] and is the basis of numerous assays for spore inactivation [8], [9]. In addition to its role as a simulant, the organism plays an important role in the biotechnology industry as a source of restriction endonucleases and of the glycosylation inhibitor nojirimycin [10].

The taxonomic placement of *B. atrophaeus* has changed dramatically over the years. Originally isolated as *B. globigii* in 1900 (Migula) as a variant of *B. subtilis*, it was originally distinguished from *B. subtilis* by the formation of a black-tinted pigment on nutrient agar and by low rates of heterologous gene transfer from *B. subtilis* [11]. It has been alternately known as *B. subtilis* var. *niger*, *B. niger*, and has been confused with *B. licheniformis* [12]. Other than the formation of the dark pigment, it is virtually indistinguishable from *B. subtilis* by conventional phenotypic analysis [13], and the lack of distinguishing metabolic or phenotypic features has contributed to the confusionin the taxonomic placement of this organism. Low interspecies DNA transfer frequencies suggested substantial divergence [11]. Based onanalysis of comparative DNA hybridization, phenotypicand biochemical tests, Nakamura advocated that pigment-producing *B. subtilis*-like isolates should be classified as a distinct species termed *B. atrophaeus* [13]. Recently, more sensitive typing methods such as amplified fragment length polymorphism analysis showed that *B. atrophaeus* strains could be classified into two major biovars: var. *globigii* encompassing the classical, commonly used BG isolates, and var. *atrophaeus* encompassing other closely related yet genetically distinct strains [14].

Here we report the definitive molecular typing of several BGstrains using whole-genome sequences, and develop a plausible microevolutionary history of a commonly used lineage based on the accumulation of mutations over time and during transfer between laboratories.The selected strains span more than six decades of development, use, and transfer of BGbetween various institutions and laboratories and offer an unparalleled opportunity to investigate mutation under selection and drift over time. Phenotypic analysis revealed substantial heterogeneity both between and within strains, even in type strains, while high-throughput metabolic profiling revealed metabolic “enhancements” to a population that had returned to the University of Wisconsin (UW) from Camp Detrick in 1952. Whole-genome comparisons of single-nucleotide polymorphisms (SNPs), small insertion/deletion motifs (indels), and large-scale genomic architecture analysis by optical maps are combined to generate a plausible history of acquisition and use of operationally relevant strains by the American Type Culture Collection (ATCC) and by several laboratories within the biodefense community.

Finally, our analysisof mutation profiles revealed potential signatures of the deliberate selection of strains with properties of enhanced growth and spore yields, properties that were deemed desirable in a simulant [6]. We also report genetic differences between strains in use in the biodefense community and the commercial sector that argue for adoption of a more uniform standard for *B. atrophaeus* as a simulant.

## Materials and Methods

### Strains and growth conditions

**Figure 1:**
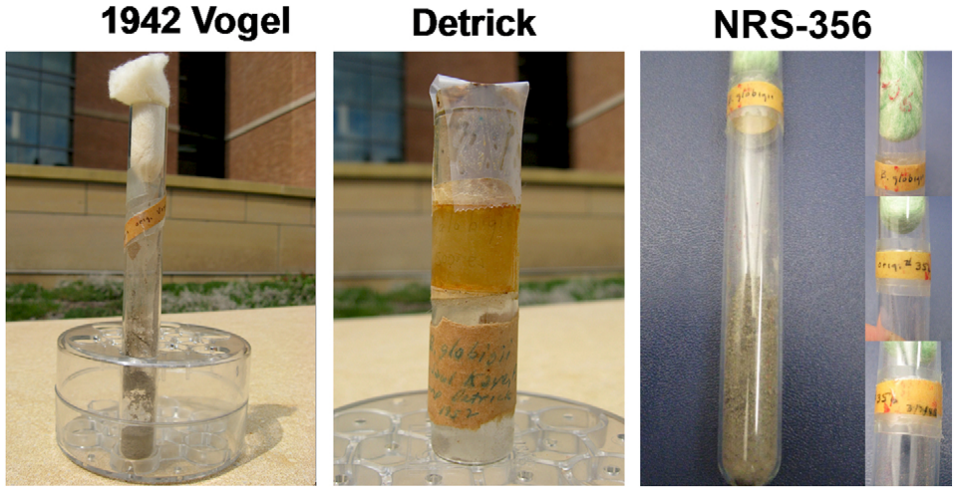
Archival samples of *B. atrophaeus* var. *globigii (“B. globigii”)* from the University of Wisconsin Department of Bacteriology. Samples had been maintained as suspensions of viable spores in sterile soil for approximately 60 years. The 1942 (left) and NRS-356 sample dated from 1944 (right) were found in the University of Wisconsin Department of Bacteriology strain collections. The 1952 sample (center) was returned to the Univ. of Wisconsin from Camp Detrick in 1952.

*B. atrophaeus* strains and their sources are indicated in Table 1. Archival strains were maintained as spores in sterile soil at the University of Wisconsin (Figure 1). The 1013 lineage, originally founded from the 1942 strain, was extensively passaged by serial transfer every 12–18 months on agar slants for 30 years. Unless otherwise indicated, strains were grown using LB agar plates, LB agar brothor Tryptic Soy agar containing 5% sheep's blood (SBA, HealthLink) at 37°C.

**Table 1:** Strains utilized in this study.

Strain	Description	Source
1942 Vogel	Archival isolate	U. Wisconsin
Detrick-1	“Camp Detrick”, morphology variant	U. U. Wisconsin
Detrick-2	“Camp Detrick”, morphology variant	U. Wisconsin
Detrick-3	“Camp Detrick”, morphology variant	U. Wisconsin
Dugway	Bioferm Lot 10–88, large-scale fermentor strain	Dugway Proving Grounds
BACI051-N	US Department of Defense reference strain, Dominant morphotype	USAMRIID
BACI051-E	BACI051, Minor morphotype	USAMRIID
ATCC 9372-1	*B. atrophaeus* var. *globigii* strain [14], derived from “Camp Detrick Red Strain”, Minor morphotype	ATCC
ATCC 9372-2	9372, Dominant morphotype	ATCC
1013-1	1942 Vogel *in vitro* passaged variant	U. Wisconsin
1013-2	1942 Vogel *in vitro* passaged variant	U. Wisconsin
ATCC 49822-1	*B atrophaeus* var. *globigii* strain, [14], Dominant morphotype	ATCC
ATCC 49822-2	49822, Minor morphotype	ATCC
NRS-356	Archival isolate	U. Wisconsin

### Analysis of colony morphology variation

Spores were germinated by plating on LB media at 37°C. Plates were examined by stereomicroscopy using indirect lighting and imaged usinga Nikon SMZ1500 with a total magnification of 16×. Colonies exhibiting distinct morphologies were repeatedly streaked to confirm stability of the phenotype.

### Whole genome sequencing

Genomic DNA was prepared from all isolates using the Blood and Cell Culture DNA Midi Kit for Bacteria (QIAGEN) from 10 ml overnight cultures in LB. BACI051-N was sequenced at the Naval Medical Research Center, while all other isolates were sequenced to >25-fold coverage at the US Army Edgewood Chemical Biological Center by massively parallel pyrosequencing on the Roche/454 GS-FLX using the Titanium reagent package. Draft genome sequences of all isolates were assembled *de novo* using Newbler [15] (Roche) and analyzed using both Newbler and Lasergene (DNAStar, Madison, WI). The 1942 Vogel isolate was designated as the reference strain and was brought to completion using standard finishing techniques.

The draft genome of *Bacillus atrophaeus* var.*globigii* was finished at the Department of EnergyJoint Genome Institute (JGI) using a combination of Illumina [16] and 454 datasets [15]. For this genome, we constructed and sequenced an Illumina GAii shotgun library which generated 15120217 reads totaling 544 Mb, which was combined with 454 Titanium standard library which generated 387327 reads totaling 137 Mb of 454 data. All general aspects of library construction and sequencing performed at the JGI can be found at http://www.jgi.doe.gov/. The initial draft assembly contained 25contigs in 25scaffolds. The 454 Titanium standard data were assembled with Newbler, version 2.3. The Newbler consensus sequences were computationally shredded into 2 kb overlapping fake reads (shreds). Illumina sequencing data wereassembled with VELVET, version 0.7.63 [17], and the consensus sequences were computationally shredded into 1.5 kb overlapping fake reads (shreds). We integrated the 454 Newbler consensus shreds, the Illumina VELVET consensus shreds and using parallel phrap, version SPS - 4.24 (High Performance Software, LLC). The software Consed [18], [19], [20] was used in the following finishing process. Illumina data was used to correct potential base errors and increase consensus quality using the software Polisher developed at JGI (Alla Lapidus, unpublished). Possible mis-assemblies were corrected using gapResolution (Cliff Han, unpublished), Dupfinisher [21], or sequencing cloned bridging PCR fragments with subcloning. Gaps between contigs were closed by editing in Consed, by PCR and by Bubble PCR (J-F Cheng, unpublished) primer walks. A total of 79additional reactions and 10shatter libraries were necessary to close gaps and to raise the quality of the finished sequence. The total size of the genome is 4 168 266 bp and the final assembly is based on 137 Mb of 454 draft data which provides an average 33.4× coverage of the genome and 544 Mb of Illumina draft data which provides an average 133× coverage of the genome. The complete sequence and WGS were deposited at DDBJ/EMBL/GenBank under accession numbers listed in Table 2. The WGS versions described in this paper are the first versions, e.g. AEFM01000000.

**Table 2:** Colony Morphology and Biochemical Data.

Strain	Morphology (LB)						Blood agar		Catalase
	Form	Elevation	Color	Sheen	Size	Margin	Hemolysis	Color	
1942 Vogel	Circular	Flat	Orange	Shiny	Normal	Undulate	++	Dark Brown	Y
Detrick-1	Circular	Umbonate	Orange	Shiny	Normal	Undulate	++	Dark Brown	N
Detrick-2	Irregular	Umbonate	Orange	Matte	Normal	Undulate	−	Beige	N
Detrick-3	Circular	Convex	Dark Orange	Shiny	Small	Undulate	+	Brown	N
Dugway	Circular	Umbonate	Orange	Shiny	Normal	Undulate	−	Beige	N
BACI051-N	Circular	Raised	Orange	Matte	Normal	Undulate	−	Beige	N
BACI051-E	Circular	Raised	Orange	Matte	Normal	Undulate	+	Beige	N
9372-1	Circular	Raised	Off-white	Shiny	Normal	Undulate	−	Grey	N
9372-2	Circular	Raised	Light orange	Shiny	Normal	Undulate	−	Grey	N
1013-1	Irregular	Raised	Orange	Matte	Normal	Undulate	−	Tan	Y
1013-2	Circular	Umbonate	Off-white	Matte	Normal	Undulate	+	White	Y
49822-1	Circular	Umbonate	Orange	Shiny	Normal	Undulate	+++	ND	Y
49822-2	Circular	Raised	Orange	Shiny	Normal	Undulate	+	ND	Y
NRS-356	Circular	Flat	Orange	Shiny	Normal	Undulate	++	Dark Brown	Y

### Identification of high-confidence mutations

Templated assembly of the remaining strains were mapped to the 1942 finished sequence using the GSMapper tool in Newbler (Roche). High-confidence mutations were selected from Newbler “HCDiffs” calls (Table S1) by applying additional selection criteria that mandated high quality scores in both reference and templated assemblies with >80% of the sequencing reads differing from the reference, elimination of mutation calls associated with homopolymer tracts (with the exception of tracts that were formed by a deletion – see below), and a minimum coverage depth of 5× with bidirectional sequence reads. Finally, the raw 454 reads from the 1942 isolate were mapped to the finished sequence to assess error bias in the 454 process and to correct for residual sequencing errors in the finished sequence. Accession numbers of the relevant whole-genome shotgun sequences are found in Table 3. Phylogeny was calculated using PAUP 4.0b10. Fifty-eightnucleotide positions were used with gaps being treated as a “5th base” and all characters assuming equal weight. One thousandbootstrap replicates were computed using a heuristic search with the optimal criterion set to “parsimony”. The tree was created using stepwise addition.

**Table 3:** Genome Sequencing and *de novo* Assembly Statistics.

Strain Name	Median Depth Of Coverage	Number Of Large Contigs	N50 Contig Size	Largest Contig Size	Percent Q40 Plus Bases	Number of Contigs	Number Of Bases	NCBI Accession number^f^	NCBI project ID
**1942** a	32^a^	n/a	n/a	n/a	99.94^a^	n/a	4168266^b^	*CP002207*	46075
**Detrick-1**	30	33	193257	809459	99.93	38	4130179	*AEFP00000000*	46077
**Detrick-2**	29	50^c^	152641	273988^c^	99.57	67^c^	4127403	*AEFQ00000000*	46079
**Detrick-3**	42	32	193246	809488	99.96	37	4131174	*AEFR00000000*	46081
**Dugway**	49	38	205337	419767	99.85	53	4130651	*AEFO00000000*	34819
**ATCC 9372-1**	44	29	255796	943057	99.91	36	4108235^d^	*AEFM00000000*	37683
**ATCC 9372-2**	42	34	250623	546945	99.88	40	4130094	*AEFU00000000*	46211
**BACI051-N**	47	78	86661	212948	99.95	81	4130502	*AEFY00000000*	51595
**BACI051-E**	36	35	192166	630535	99.97	42	4130076	*AEFX00000000*	48615
**1013-1**	31	30	282215	914719	99.95	39	4130375	*AEFS00000000*	46207
**1013-2**	30	33	225488	512878	99.93	40	4057611^e^	*AEFT00000000*	46209
**ATCC 49822-1**	37	35	192016	638113	99.92	50	4132165	*AEFV00000000*	46283
**ATCC 49822-2**	34	34	213362	638120	99.89	51	4135194	*AEFW00000000*	46285
**NRS-356**	44	42	178925	527969	99.97	44	4128298	*PENDING*	*PENDING*

a Genome was finished and closed to a single contig. Depth of coverage and Q40+ metrics are for *de novo* assembly of 454 data only.

b 454Draft sequences appear smaller than 1942 due to collapse of repeat regions.

c The average read length of this sample was considerably lower (239 versus >300) for other genomes in this dataset, resulting in larger number of contigs and decreased assembly quality.

d Deletionof 23 kb with clear join points (positions 4,022,138–4,045,817) verified by optical mapping (Figure 4B).

e Deletion of 73 kb with clear join points (positions 3,992,613 to 4,065,341) verified by optical mapping (Figure 4B).

### Confirmatory sequencing of SNP/Indels

Nineteen loci in which putative mutations were identified from the 454 dataset were re-sequenced from PCR products by standard Sanger dye-terminator methods. No false-negatives or false-positives were identified among the re-sequenced loci; however resequencing of the apparent mutation at position 1486408revealed mixed genotypesin several isolates that are artifacts of a large duplication in the 1942 chromosome. Therefore, this signalcannot be considered a true SNP.

### Annotation, comparative genomic analysis, and multiple alignments

Preliminary annotations were generated using a combination of the RAST [22] algorithm (rast.nmpdr.org). Loci containing mutations were used to query the non-redundant (nr) databases and Refseq protein databases at NCBI using directed BLASTx and BLASTp. The comparative BLAST tool from RAST was utilized for genome-wide protein sequence comparisons to *B. subtilis*. Results were filtered for bi-directional hits. Multiple alignments were generated by MegAlign from the LaserGene software package using the CUSTALW algorithm.

### Optical mapping

Genomic DNA was prepared from live bacteria on agar slants to maximize the yield of extremely high-molecular weight DNA. Optical maps were generated by digestion with *Nco*I of DNA arrayed linearly on glass slides and the resulting maps were aligned and compared with the MapSolver software package (OpGen, Inc., Gaithersburg MD).

### Information-based Genomic Distance (IBGD) analysis

Using an information-based method for genomic classification [23], the sequence contigs from BG isolates 1942, 1013-2 and 49822 were analyzed in order to map the phylogenetic relationships of these isolates to other *Bacillus* species. In this method, genomic content is characterized by the frequencies of occurrence of short n-mers contained within each sequence (n typically from 3 to 16). These n-mers are then rank ordered by genome. The pair-wise comparison of the rank of n-mers within two different genomes is then used to compute an information-based genetic distance (IBGD), where the sum of the differences in rank for all possible n-mers is weighted by an entropy factor that depends on the frequencies of occurrence of the respective n-mers in the two genomes. The pair-wise IBGD values are then used to construct a phylogenetic network [24]. *Bacilli* genomes were obtained from Genbank. This method for phylogenetic characterization enables computation even with the unassembled reads, and it can be applied to draft or partial genome sequence data, which was the case for the three *B. atrophaeus* genomes studied here.

### Phenotype microarray (PM) analysis

The first seven BGstrains listed in Table 1 were streaked for single colonies on BHI plates and incubated at 33°C overnight, followed by subculturing a second time under the same conditions. Subsequently, cell suspensions were prepared according to Biolog specifications, with OD readings ranging between 0.35–0.45 at 600 nm. Biolog phenotypic microarray plates PM1 through PM20, were inoculated according to the manufacturer's specifications, and incubated at 37°C for 72 hours. Readings were taken every 15 minutes, and data processed by OmniLog Phenotype Microarray File Management/Kinetic Plot and Parametric modules. Two biological replicates of the experiment were conducted for each strain. PM1-10 contain single wells for each growth condition whereas PM11-20 contain quadruplicate wells for each condition.

### Statistical analyses and heatmap of phenotype microarray data

The area under the curve (AUC) values were computed by adding all OmniLog values at all time points for each of the 1200 distinct phenotypes produced from the OmniLog software. The AUC values from the two different biological replicates for each unique phenotype were averaged. The ratio for each AUC was calculated between the 6 query strains (Detrick-1, Detrick-2, Detrick-3, 1013-1, 1013-2, and Dugway) and reference parent strain (1942). For the purpose of visualization, 1920 phenotypes were included in the heatmap (i.e. this better represents the locations of the phenotypes which correspond to different modes of action categories). The same ratios were used for the phenotypes that have replicates. The ratio values were formatted as PM1 to PM20 for each strain across the columns and wells A*_i_* to H*_i_*, where *i* = 1 to 12 for the rows. The results were plotted in a heatmap using R [25]. Positive growth wells are represented by greenblocks while negative growth wells are represented by red blocks.

### Catalase assay

Catalase activitywas assayed by spotting drops of hydrogen peroxide (3%) onto isolated colonies on LB agar plates. Colonies were monitored for bubble formation, signifying the release of water and oxygen. A colony was considered to be catalase positive by observation of bubbles.

### Sporulation efficiency assays

Streaks of Detrick 1, Detrick 2, and 1013 strains were grown for two days on TSA plates containing SBA.Bacterial cell mass was scraped using an inoculating loop (1 µl) from the streak and resuspended in PBS. Sporulation was evaluated by bright field phase-contrast microscopy. Phase-bright free sporesand phase-dark vegetative cellswere counted. Five representative viewing fields were counted from each strain for each experiment. This experiment was completed in triplicate by repeating once per day over the course of three consecutive days.

In order to compare the percent sporulation between Detrick 1 and Detrick 2, and Detrick 1 and 1013, a mixed analysis of variance (ANOVA) was used to complete the analysis. Strain and viewing field were evaluated as fixed factors, and replicate was included as a random factor. The natural log of the percent sporulation was taken to obtain a normal distribution of the residual error. Tukey's method was applied to compare the difference between the mean log percent sporulation.

## Results

### Historical investigations of BG provenance

We traceda potential provenance of the commonly used BGstrains through an exhaustive search of the open literature and the archives of the University of Wisconsin,which suggested a possible lineage from which the “military” BGstrains were derived. The original source of the strains were the collections at the University of Wisconsin during the 1930s and 1940s, from which the strains were transferred to Camp Detrick at the initiation of the US Army's BW program at the beginning of the Second World War [6], [26]. At Camp Detrick, BG was used as a non-pathogenic surrogate in process development for spore-forming bacteria It is tempting to speculate that the University of Wisconsin supplied BG to Porton Down: A note found in the archive of Dr. Baldwin's papers, dated February 19, 1943, contained an order from Dr. Fildes (presumably Sir Paul Fildes, a noted bacteriologist active in the British BW program at the time), for a batch of *B. subtilis* spores. It is not clear whether BG or *B. subtilis* subsp. *subtilis* was supplied, or whether this material was actually delivered. Unfortunately, original records describing in detail the maintenance of the strains during the period 1942–1955 were destroyed as per US Army policy at the time (Dr. Mark Wolcott, USAMRIID; personal communication), and the personnel who had first-hand knowledge of the strain passage histories and methods are deceased. Therefore, the actual source of the Camp Detrick isolates must be inferred from published work [6], limited available documentation (e.g. ATCC 9372) and the genome sequences presented hereFrom Camp Detrick the isolates were eventually transferred to ATCC as *B. subtilis* var. *niger* “red strain.” The desire to maintain a phenotypically and genotypically uniform simulant throughout the biodefense communityprompted us to elucidate whether significant phenotypic and/or genomic differences had accumulated in any of the commonly used isolates during the growth and transfer of strains to different institutions and to compare the isolates in broad use today to the so-called “Mil-Spec” strain (ATCC 9372).In contrast, the origin of ATCC 49822 prior to acquisition F. Young's laboratory (the depositor) is unclear.

We obtained isolates from archival spore suspensions in sterile soilfrom the University of Wisconsin with legible labels dating back as far as 1942 (Figure 1; Table 1). These isolates included an archival stock dated 1942 that likely predated the transfer to Camp Detrick, as well as material that had been returned to the University of Wisconsin from Camp Detrick in 1952. A derivative of the 1942 strain that had been repeatedly passaged *in vitro* on agar slants over a period ofapproximately 30 years allowed us to compare the genomic signatures of deliberate selection with the effects of long-term *in vitro* passage. In addition, a sample of strain NRS-356 [13], which is mentioned as a possible parent strain in correspondence between various academic laboratories and Camp Detrick, was also obtained from the same source as the 1942 “Vogel” strain. These isolates were subsampled, germinated on LB plates, screened for colony morphology variation (see below). Genomic DNA was prepared from these isolates for sequencing.

### BG strains exhibit distinct colony morphologies

**Figure 2:**
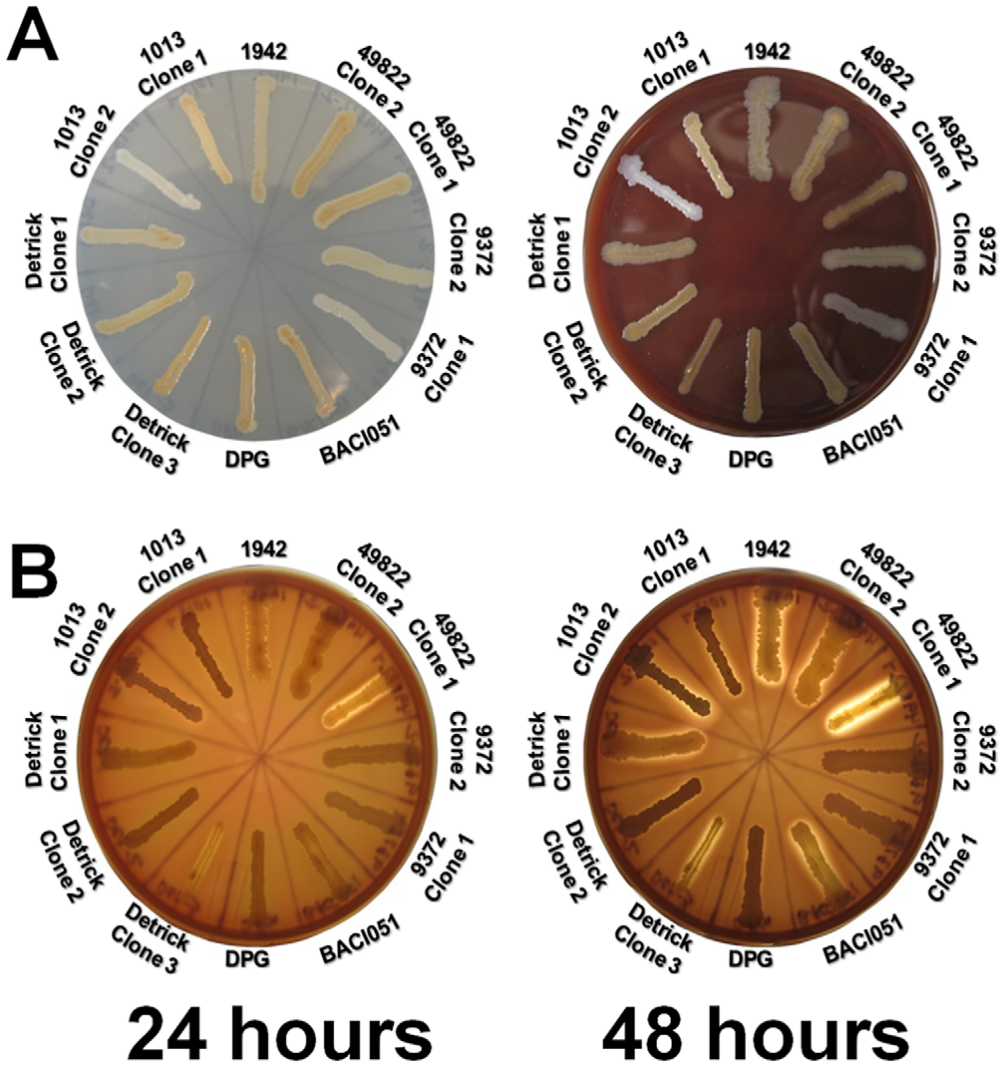
Appearance of *B. atrophaeus* strains on solid media. **A**) Appearance of *B. atrophaeus* strains on LB or blood agar plates after 24 hours at 37°C. Plates were illuminated directly. **B**) **β**-Hemolysis of some *B. atrophaeus* strains. Transilluminated plates after 24 or 48 hours of growth on blood agar at 37°C.

Upon initial plating of the archival and modern-dayBG stocks, we noted distinct colony morphotypes for many of the strains, with some strains containing multiple variants (Figure 2, Table 2). Some of these morphotypes were consistent with those observed by Hayward *et al.* [6] whooriginally described the emergence of colony variants in “*B. globigii*.” As in the earlier report, individual morphotypes were stable and did not interconvert with high frequency (data not shown), suggesting that these morphotypes were the result of relatively rare chromosomal mutations, although 1013-1 occasionally threw off papillae in heavier streaks (not shown). Multiple morphotypes were noted for ATCC9372, ATCC 49822, Detrick, and 1013, while the archival 1942 isolate, the isolate obtained from Dugway Proving Ground (Dugway) and BACI051 appeared to be pure populations on LB. All strains tested positive for BGusing Real Time-PCR primers specific to the *recF* gene (Methods S1) [27]. The appearance of multiple colony morphotypes even within single “strains” strongly suggested an as-yet undescribed level of genetic diversity within these samples that likely affected the expression of cell-surface components and/or sporulation. The intra-strain colony morphology variation was particularly dramatic in the *in vitro* passaged 1013 and ATCC9372 isolates, in which one variant of each lineage had lost the production of color on LB orSBAplates (Figure 2), suggesting more dramatic alterations to the genome.

### Whole genome sequencing of BG isolates

Draft genome sequences were generated from several BGstrains in our collection. A summary of the results from the sequenced isolates is indicated in Table 3. All of the “military” isolates (Detrick clones1through 3, BACI051, Dugway) were extremely closely related to each other and to both ATCC9372 variants. The ATCC isolates possessed additional mutations that were absent in the “military” isolates. The size of the finished and closed genome of *B. atrophaeus* var. *globigii* 1942 was 4,168,266 bp, and annotation using RAST [22] revealed 4433 features, including 4343 protein-coding sequencesand 90 RNA molecules [28]. The preliminary annotations derived from RAST are available as Genbank .gbk files in the supplementary material.

### Bioinformatic analysis of sequence data

**Figure 3:**
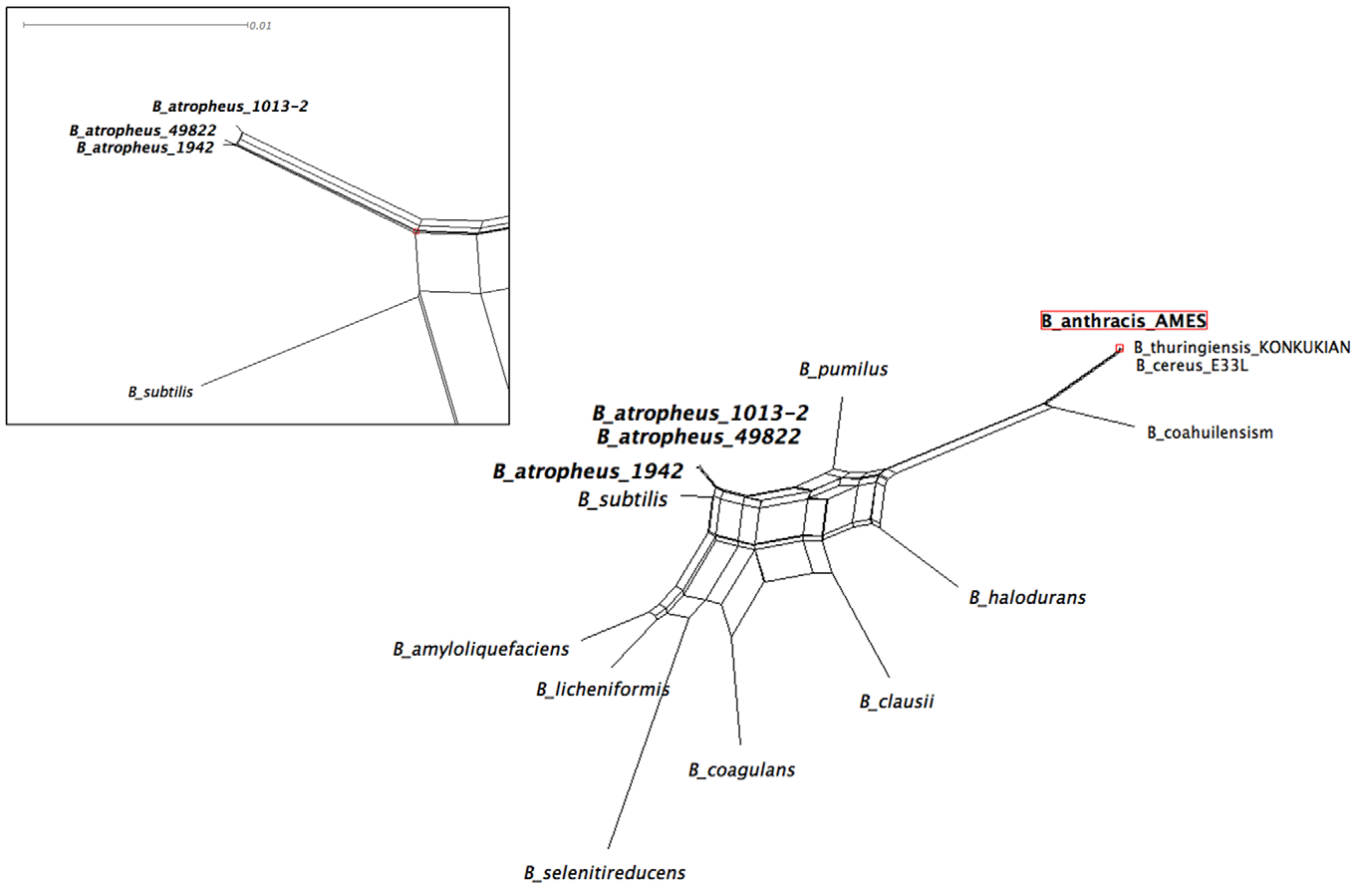
Identification of *B. subtilis* as nearest-neighbor to *B. atrophaeus* var. *globigii* by whole-genome phylogenetic analysis of *Bacillus* genomes. Information-based genomic distance (IBGD) was determined by comparing the relative distributions of n-mers within each genome to generate a pair-wise matrix of relative n-mer frequencies (see Materials and Methods). Variation of the n-mer length between 4 and 8 did not substantially affect the derived phylogeny. In this case n-mer length of 5 was utilized. For clarity, only three select species of the *B. cereus* group (of more than 30 that all cluster together) are labeled on the figure. The apparent divergence of isolate 1013-2 is due to alteration of the n-mer frequencies as a result of the deletion of 72 kb of genomic material.

On average, the genome of *B. atrophaeus* is approximately 86% identical to *B. subtilis* on the nucleotide level,supporting its delineation as a distinct species and agreeing well with previous estimates [29]. Analysis of the IBGD using whole-genome sequences (N-mer length >4) supported the identification of *B. subtilis* 168 as the closest relative among sequenced bacterial genomes (Figure 3). For this particular case, n = 5 (i.e., there were 4^5^ = 1024 total 5-mers used to compute the IBGD). The IBGD values were relatively insensitive to the choice of n over the range of 4–8. Thethree BGgenomes analyzed grouped closely together, and our analysis of the *Bacillus*-wide phylogeny using IBGD revealed the phylogenetic distance of that *B. subtilis/B. atrophaeus* species from *B. anthracis*, supporting the inferences published elsewhere from rRNA sequence analysis (Figure 3) [30]. Primary amino acid sequences of RAST-annotated proteins are on average 72% (median 83%) identical between *B. atrophaeus* and *B. subtilis*. When only the proteins that yielded bidirectional BLAST hits in RAST are examined, the predicted proteome of *B. atrophaeus* is, on average, 83% identical (86% median) to *B. subtilis*.

We utilized the finished sequence of the 1942 isolate as a reference strain for templated assembly of the remaining BG draft sequences. Two additional ATCC isolates of *B. atrophaeus *(49337 and 6537) were distinguishable from var. *globigii* on the basis of very high SNP/indel counts, lower coverage ofand percentage of reads mapping to the 1942 reference, and unique genomic features which supported their proposed classification as var.*atrophaeus* [14]. The distinguishing genomic features of var.*atrophaeus* strains and the delineation of the *B. atrophaeus* clade from *B. subtilis* will be published elsewhere.

### Scaffolding of “military” BG genomes using optical maps

**Figure 4:**
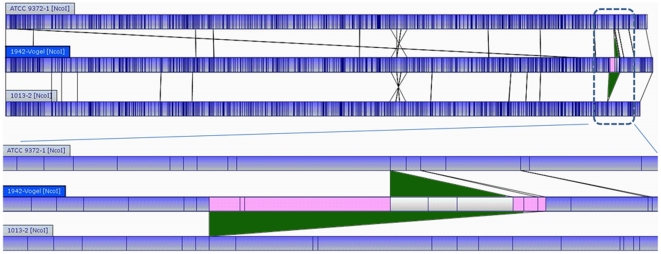
Optical mapping of *B. atrophaeus* var. *globigii* and detection of a 72 kb deletion. **A**) Whole-genome consensus optical restriction maps (*Nco*I) of *B. atrophaeus* ATCC 9372-1 (Top), 1942 (middle) and 1013-2 (bottom) isolates. **B**) expanded view showingdetail of the deleted regions in ATCC 9372-1 and 1013-2.

Optical restriction mapping [31], [32], [33] was used to compare the overall genomic structure of selected isolates. No differencesin overall genome architecture between the “military” BG isolates, the archival 1942 isolate, or 1013-1 were observed (Figure 4, data not shown), suggesting that the global architecture of these isolates is relatively stable, even over 30 years of serial *in vitro* passage.However, the optical maps and sequence coverage analysis of 1013-2 and 9372-1revealed substantial deletions of approximately 72,727and 23,678 bases, respectively, of genomic materialspanning from positions 3,992,613 to 4,065,341 (1013-2) or 4,022,138–4,045,817 (ATCC 9372-1) (Figure 4; Table S2). The genes within this deleted region are listed in Table S3 but notably contain genes encoding for nitrite reduction, germination (*gerKABC*), and biosynthesis of the lipopeptide surfactin (*srfCAB*) [34], [35]. A defect in surfactin production is a particularly intriguing candidate for the morphology and pigmentation variations in 1013-2 and ATCC 9372-1, since disruption of *srfA* has been shown to have dramatic effects on spreading motility on semisolid media, on biofilm formation [34], [36], and low-grade hemolytic activity.

### Mutation analysis of BG isolates

**Figure 5:**
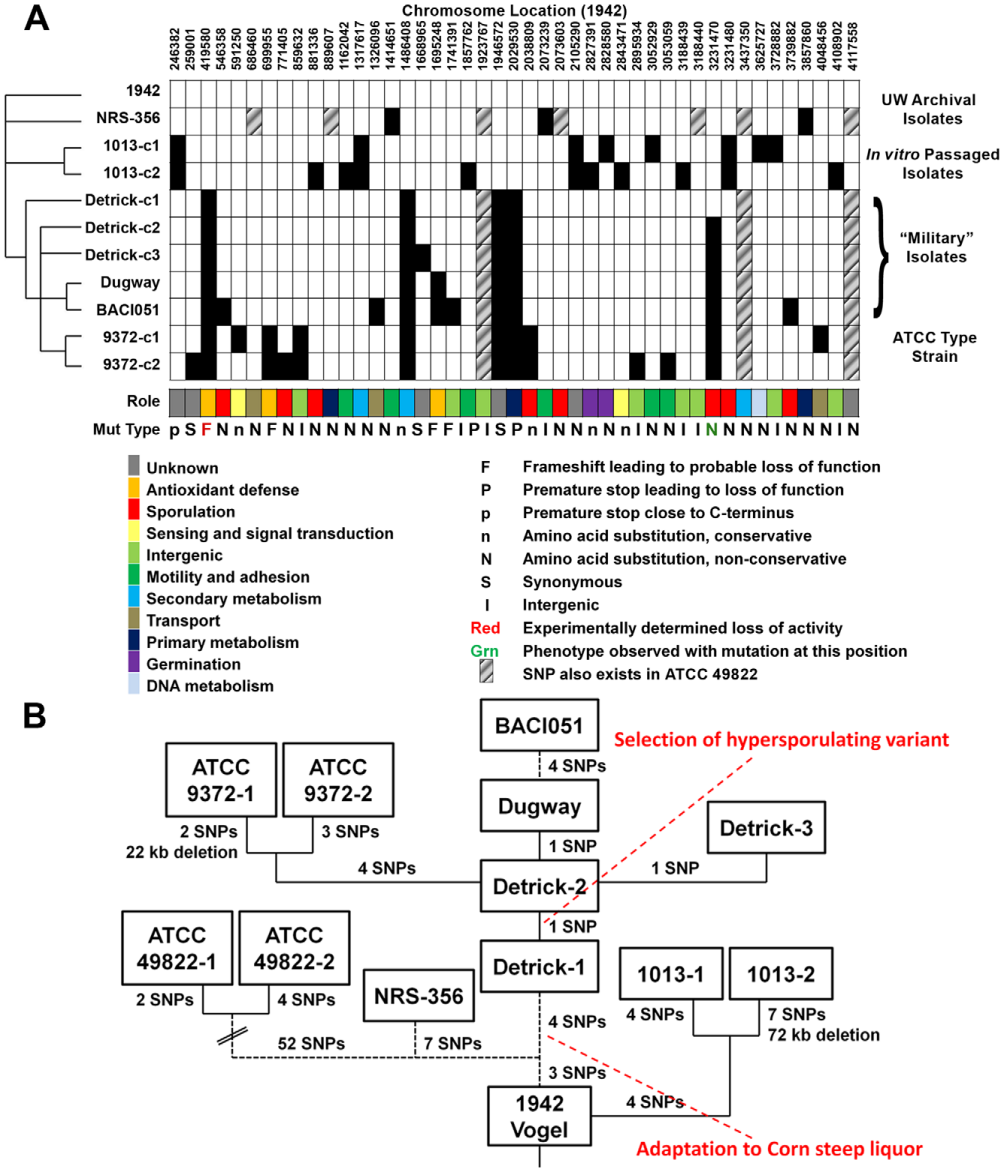
Whole-genome mutation analysis and evolutionary history of the “military” lineage of *B. atrophaeus* var. *globigii.* **A**) Whole-genome shotgun sequences of the other strains were mapped to the *de novo* assembled contigs of the 1942 strain using Newbler. Mutations exhibiting high quality scores in both reference and query sequences and with differences from the template exhibited in >85% of the individual sequencing reads are indicated as a blackened box. In one case (position 259001 in ATCC 9372-1) an initial false-negative due to the formation of a homopolymeric tract was found by direct inspection of the assemblies. The genes whose functions are altered by the given mutation are indicated in Table 4. **B**) Microevolutionary history of *B. atrophaeus* var. *globigii* strains. “Enhancement” events are indicated in red.

Using the *de novo* assembled draft sequence from the 1942 isolate as a template for subsequent analysis of SNPs and small indels in the other “military” isolates, we generated a list of high-confidence, discriminatorymutations that differentiate the strains (Figure 5A). The nature and annotation of the mutations are found in Table 4 and can be assigned an approximate temporal order in which they occurred (Figure 5B). Based on this analysis, 1942 is the most likely parental strain for all of the isolates in this study, with the 1013 lineage diverging earliest, followed by 49822, then the “military” lineage prior to the transfer to Camp Detrick. This conclusion is based on the observation that 49822 shares three SNPs with Detrick-1. The latter is the most likely progenitor of the other “military” isolates, since it has the fewest mutations relative to strain 1942. Detrick-1 can be differentiated from other “military” isolates by possessing the parental allele of *spo0F* rather than the H101R allele (position 3231470) that is characteristic of all of the other “military” BGisolatesand the ATCC9372 strains. The two colony morphology variants of ATCC9372 each exhibited distinct mutation profiles indicating that the reference strain is in fact a mixed population of at least two genetically distinct substrains.

**Table 4:** SNP/Indel locations in *B. atrophaeus* genomes^1^.

Genome location	RAST Locus (NCBI Locus Tag #)	Types^2^	Gene affected^3^	*B. subtilis* Homolog (% Identity)	Mutation	AA change	Position in protein/total length
246382	BG236 (01550)	PS	*yetF* – hypothetical protein	BSU07140 (73.9)	C:T	*	217/231
259001	BG250 (12250)	SYN	*yfnL* – hypothetical protein	BSU07230 (91.5)	C:T		
419580	BG405 (02000)	1 bp INS	*katA* – vegetative catalase	BSU08820 (94.4)	+T		197/483
546358	BG536 (02620)	NS	*hpr/scoC* – MarR family regulator(extracellular protease expression and sporulation)	BSU1000 (85.7)	C:G	A:P	13/200
591250	BG584 (02840)	NS	*hemAT* – heme-based aerotactic transducer	BSU10380 (87.7)	C:T	R:K	215/432
686460	BG685 (03350)	NS	Oligopeptide ABC transporter, periplasmic oligopeptide-binding protein OppA	BSU11430	T:G	W:G	520/545
699955	BG706 (03425)	PS	*yjbL* – truncated hemoglobin	BSU11590 (96.1)	+C	*	89/132
771405	BG795 (03855)	NS	*rapA* – response regulator (Dephosporylates Spo0F)	BSU12430 (91.2)	T:G	S:A	261/378
859632	BG904/905 (04400/04405)	IG			T:C		
881336	BG929 (04515)	NS	*kinE* – 2-component histidine kinase; regulates Spo0F activity	BSU13530 (84.6)	C:T	P:L	577/739
889607	BG939 (04560)	NS	Methylthioribulose-1-phosphate dehydratase (EC 4.2.1.109), *mtnB*	BSU13610 (87.6)	A:G	Y:C	86/209
1162042	BG1225 (05960)	INS	*fliY* – flagellar switch motor protein	BSU16320 (87.2)	+GTGGTC	V_3_:V_5_	355/379
1317617	BG1320 (06415)	NS	*pksR* – Polyketide synthase	BSU17720 (71.0)	G:C	C:S	1698/2574
1326096	BG1327 (06450)	NS	*ebrB* – multidrug resistance family protein	BSU1729 (77.8)	A:G	S:P	24/115
1414651	BG1226 (07000)	NS	Chemotaxis regulator - transmits chemoreceptor signals to flagelllar motor components CheY	BSU17940 (86.0)	C:T	A:V	97/120
1486408^4^	BG1487 (07220)	SYN	*ppsA* – Plipistatin synthase/non-ribosomal peptide synthetase.	BSU18340	G:A		1872/2560
1668965	BG1681 (08240)	1 bp DEL	*yozB* – putative inner membrane protein	BSU19140 (93.8)	−T		55/178
1695248	BG1706 (08360)	5 bp INS	*yojO* – hypothetical protein, potential nitricoxide synthase activation protein	BSU19380 (87.5)	+GCTCT		140/638
1741391	BG1764/1765 (08650/08655)	IG			A:G		
1857762	BG1903 (09335)	PS	*ypfA* – possible pilZ homolog	BSU22910 (60.1)	G:A	*	197/218
1923767^5^	BG1978/1979 (09750/09755)	IG			C:T		
1946572	BG1997 (09870)	SYN	Putative phage protein	NA	T:C		283/584
2029530	BG2096 (10375)	PS	2-methylcitrate synthase	BSU24140 (78.3)	+GA	*	197/218
2038809	BG2105 (10420)	NS	*spo0A* – 2-component response regulator, controls initiation of sporulation	BSU24220 (96.6)	G:A	A:V	225/265
2073239	BG2144/2145 (10615/10610)	IG	Intergenic - possible promter region of *sinI* gene (repressor of SinR)		T:C		
2073603	BG2146 (10620)	NS	SinR, regulator of post-exponential-phase responses genes (competence and sporulation)	BSU26100 (98.0%)	A:G	K:E	
2105290	BG2186 (10820)	NS	*yqgE* – putative efflux transporter	BSU25010 (92.5)	G:C	H:D	390/430
2827391	BG2944 (14450)	NS	*gerAB* – spore germination protein	BSU33060 (67.7)	G:A	R:K	35/365
2828580	BG2945 (14455)	NS	*gerAC* – spore germination protein, governs germination in response to alanine	BSU33070 (61.8)	G:T	D:Y	64/372
2843471	BG2960 (14530)	NS	*yvrG* – sensor histidine kinase involved in cell wall processes	BSU33210 (75.4)	T:G	I:L	18/580
2895934	BG3014/3015 (14785/14790)	IG			A:G		
3052929	BG3176 (15575)	NS	Flagellin	NA	A:G	I:T	279/320
3053059	BG3176 (15575)	NS	Flagellin	NA	C:T	A:T	236/320
3188439	BG3318/3319 (16270/16275)	IG	Intergenic		10 bp deletion		
3188440	BG3318/3319 (16270/16275)	IG	Intergenic	NA	T:G		
3231470	BG3372 (16525)	NS	*Spo0F* – 2-component response regulator. Integrates signals from sensor kinases to trigger entry into sporulation	BSU37130 (98.4)	T:C	H:R	101/124
3231480					C:G	A:P	98/124
3437350^5^	BG3610 (17625)	NS	Short chain dehydrogenase/reductase	NA	T:C	T:A	149/247
3625727	BG3807 (18590)	NS	*trmE* – GTPase and tRNA-U34 5-formylation enzyme TrmE	BSU41020 (93.3)	C:A	D:Y	2/459
3728882	BG3897/3899^6^ (19040/19045)	IG			AA:TT		
3739882	BG3911 (19100)	NS	*sigH (spo0H)* RNA polymerase sigma factor	BSU00980 (98.6)	C:T	P:L	197/218
3857860	BG4037 (19695)	NS	Glycosyltransferase	NA	T:C	G:R	374/402
4048456	BG4209 (20515)	NS	*yclF/dtpT* – putative di-tripeptide-proton ABC transporter	BSU3670 (87.0)	A:G	W:R	397/492^7^
4108902	BG4273/4274 (20825/20830)	IG			C:A		
4117558^5^	BG4285 (20875)	NS	*ydaL* – uncharacterized membrane protein	BSU04290 (76.5)	C:T	P::L	9/574

1 In addition to the above mutations, putative SNPs at positions 39912, 45501, 61413, 611822, 1170114, 1251549, 2174033, 2771980, 3128882, 3269962, 3354410, 3528224, and 3880949 were common to all reference mapping experiments against the finished genome using 454 data, including the 1942 dataset. The commonality to all datasets suggest that these were errors in the finished sequence that are being verified.

2 PS = Premature stop; SYN = Synonomous; INS = Insertion; NS = Non-synonomous; IG = Intergenic; DEL = Deletion.

3 Annotations are a combination of RAST and directed tBLASTn and BLASTp searches vs *Bacillus* databases.

4 Forms part of a large polypeptide synthase containing highly homologous regions.

5 Also shared with strain ATCC 49822.

6 The conserved start codon of the *radA* gene (BG3899) of *B. atrophaeus* and *B. subtilis* falls within the BG3898 ORF. Therefore BG3898 as called by RAST is not deemed likely to be a protein-coding gene.

7 In putative transmembrane region.

### Effects of genotype on strain phenotypes

The 72 kb deletion in 1013-2 included the structural genes for biosynthesis of surfactin, a cyclic lipopeptide with a mild hemolytic activity [34]. To test whether the “military” and *in vitro* passaged strains possessed low-grade hemolytic activity, we streaked these variants on rich agar media containing 5% sheep's blood and looked for hemolysis. To our surprise, all strains exhibited striking variation in their coloration (Figure 2A), with the 1942, 9372-1 and Detrick-1 isolates considerably darker on blood agar than the other “military” and *in vitro* passaged isolates. In addition, on LB the 1013-2 and 9372-1 isolates appeared white and off-white, respectively. Pigmentation of *B. subtilis* colonies is associated with production of a melanin-like pigment by the CotA protein, a major component of the spore coat [37]. In addition to the variations in pigmentation, streaks of the 1942 and Detrick-1 isolates were consistently translucent under transillumination (Figure 2B). These zones of translucency are suggestive of weak β-hemolysis, which has previously been observed in *B. subtilis* strains that produce high levels of surfactin [34], [35], [38]. The other strains exhibited either weak α-hemolysis or none at all, with the exception of the strongly hemolytic 49822-1 variant. At least in the “military” lineage, the quasi-hemolytic phenotype and dark-brown colony pigmentation correlated with the presence of a wild-type *spo0F* allele, suggesting that the ability of *B. atrophaeus* to lyse red blood cells may be regulated in part by *spo0F*. However this was not universally the case; the BACI051 strain had two discernible variants on SBA (not shown), one of which appeared to have recovered partial hemolytic activity (Figure 2B).

### BG strains have distinct metabolic profiles

**Figure 6:**
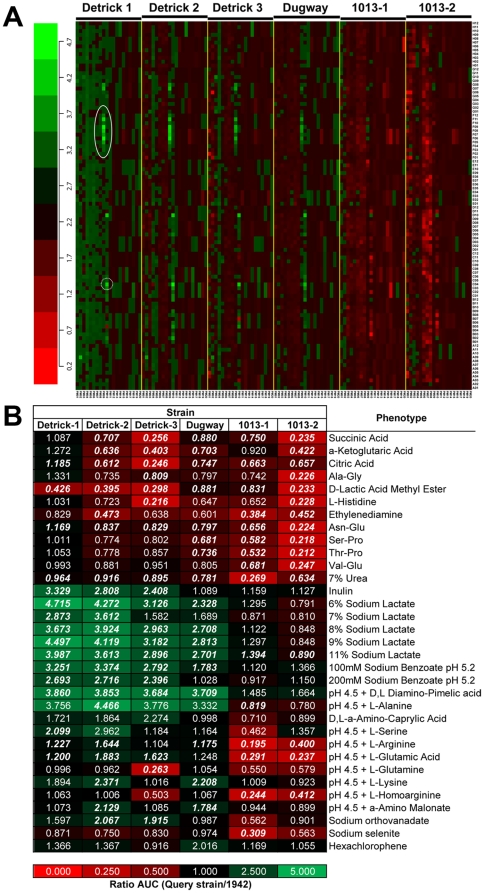
Omnilog phenotypic arrays of *B. atrophaeus* subsp. *globigii* strains. Six strains were each inoculated into twenty 96-well Omnilog plates and grown at 37°C. Reduction of tetrazolium dye by respiring cells was measured every 15 minutes by optical density. Dye reduction relative to the 1942 strain is shown; the red ratio values indicate less respiration while the green ratio values indicate more respiration as compared to the 1942 strain. Individual arrays or strains are displayed in each of the six major columns labeled Detrick 1, Detrick 2, Detrick 3, 1013-1, 1013-2, and Dugway. **A**) *Heat map of all conditions for each strain*. Each of the twenty plates for each strain is represented by the notation PM01-PM20 (left-to-right for each strain) along the x-axis. The rows represent the well position, and are denoted as A_i_ to H_i_ (i = 1 to 12) from the bottom to the top of the plot in each array along the y-axis. Each cell ratio value represents the average of two biological replicates for each strain. Plates PM01-PM10 contains single wells for each growth condition, while plates PM11-PM20 contain quadruplicate wells for each growth condition. Solid circle indicates wells containing sodium lactate; dotted circle indicates well containing L-serine at pH 4.5. The details of the 1920 growth conditions can be found in the first worksheet labeled “All strain AUC data” in Table S4. **B**) Most significant phenotypes for each of the six test strains as compared to the 1942 strain. The phenotypes with statistically significant increases and/or the decreases in ratio values for each of the six strains are presented. For the 1013 isolates only the conditions giving the five largest changes are presented. The number in each color block indicates the ratio for the test strain relative to the parent strain for the phenotype specified. The details of all significant phenotypes for each test strain can be obtained in Table S4. *Bold Italic* font indicates p<0.05.

To gain insight into the effects of genetic divergence of the adapted isolates on their metabolic capacity, the Detrick isolates and the separate 1013 isolates were compared by multiphenotype analysis using the Omnilog system, which allows the high-throughput comparison of 96×20 discrete growth conditions, including carbon, nitrogen, phosphorus, sulfate, nutrient supplements, pH, osmolytes as well as a broad class of growth inhibitors. The growth of the 1942 strain was used as a reference for determining relative growth rates of the other strains. The results of these experiments are summarized in Figure 6 and Table S4. In general, growth of the 1013 isolates was significantly diminished relative to the 1942 in many different growth conditions, most notably in the ability to use amino acids and peptides as carbon and nitrogen sources, to withstand osmotic stress, and to grow under reduced pH. In addition, the strains had developed sensitivity to beta-lactams, quinolones, and membrane-disrupting activities. These results suggested broad combined effects of several mutations on the phenotype of the strains. In addition to the *spo0F(A98P)* allele, which is a likely candidate for highly pleiotropic effects on the decision to sporulate under many different conditions, both strains contain substitutions in the *yetF* and *yqgE* genes that may be contributing to the phenotypes observed. The more pronounced defect in 1013-2 may be attributable to defects in the *gerAB* and *gerAC* genes and the large 72 kb deletion which contains several genes involved in germination.

By contrast, the Detrick isolates in general grew more robustly than the 1942 strain under multiple growth conditions. Increased relative growth rates were particularly pronounced for acidic conditions and media containing osmolytes, but particularly for wells containing sodium lactate [6].

Another isolate in the “military” lineage, Dugway, is clearly derived from the Detrick lineage by SNP/indel profiling yet has a metabolic profile that is much closer to the parental strain. Like the Detrick isolates, the Dugway strain grows better at low pH, but many of the other conditions do not promote elevated growth relative to 1942. Only one mutation differentiates that isolate from the Detrick-2 isolate – a 2-bp insertion in the *yojO* gene encoding a putative activator of nitric oxide (NO) synthesis. Again, the physiological role of this mutation is unclear, although nitric oxide synthesis plays a critical role in modulating antibiotic resistance in *Bacillus* spp. [39]. In addition to its role in promoting resistance to antibacterial drugs, NO is known to modulate *B. subtilis* genes involved in nitrate respiration when oxygen is limited [40]; thus the lowered growth in this strain may reflect the inability to grow to higher densities and overcome the resulting lower oxygen tension. An additional isolate, BACI051 is clearly derived from Dugway, yet two variants have accumulated additional mutations in *sigH (spo0H)*, *hpr/scoC*, and *ebrB*. Notably, the phenotype of BACI051-E on plates more closely resembles the 1942 strain (Figure 2B).

### Catalase activity of BG

sequencing of the “military” isolates revealed a frameshift mutation in the *katA* gene encoding the major vegetative catalase [41]. The absence of catalase activity in “military” isolates was confirmed by adding a solution of 3% H_2_O_2_ to smears of various strains. In contrast to the 1942 strain, which exhibited immediate and robust catalase activity, the strains containing the frameshift lacked this activity. A small amount of bubbling could be seen, probably due to the presence of a second catalase normally packaged in spores [42].

### Sporulation efficiency

**Figure 7:**
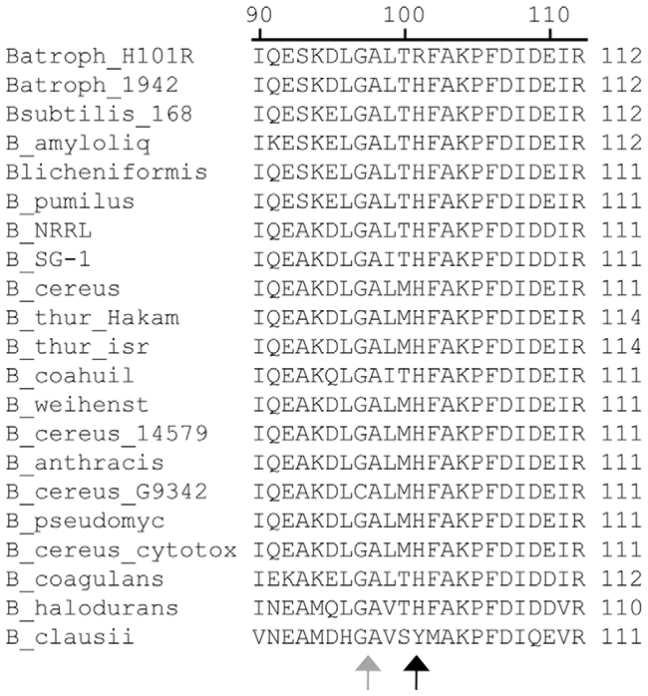
The *spo0F(H101R)* and *spo0F(A98P)* alleles are associated with hypersporulation. Phase-contrast microscopy of BG strains after two days of growth on SBA. Vegetative cells appear as phase-dark rods, while spores appear as round, phase-bright globules. The mean percentage sporulation of each strain in a representative experiment is given ±SEM. The experiment was repeated on three consecutive days; representative results of a single experiment are shown. Statistical significance was determined by mixed ANOVA (Tukey's method, p<0.05).

To test whether the phenotype observed on blood agar was associated with differences in sporulation, selected strains were grown for two days as patches on blood agar, resuspended in PBS and counted directly. Strain Detrick-2exhibited significantly higher percentages of phase-bright spores than the Detrick-1 strain (Figure 7, Mean +/− standard error of the mean). Similar results were observed for the 1942 and Dugway strains (not shown). The 1013-1 strain exhibited an even higher degree of sporulation than the Detrick-1 strain under identical conditions (Figure 7).

## Discussion

*Bacillus atrophaeus* has historically been grouped with *B. subtilis*, and is usually described as a black-pigmented variant (var. *niger*) because of its many phenotypic similarities to the better-characterized *B. subtilis*. Both organisms are soil-dwelling, non-pathogenic saprophytes, but have been differentiated by the ability to produce pigment on nutrient media containing an organic nitrogen source [13]. The orange pigmentation of *B. atrophaeus* var.*globigii* spores made it an attractive simulant for *B. anthracis*, facilitating the detection of dispersed spores in complex environmental samples. Recently, more sensitive phylogenetic approaches using AFLP have delineated *B. atrophaeus* as a separate species [13], [14]. The taxonomic confusion has arisen due to inadequately sensitive typing methods, and has led to misattribution of pathogenic qualities associated with some *B. licheniformis* strains to the *B. atrophaeus* strains currently in use as simulants [12], for which no direct evidence of pathogenicity exists. This report defines the genomic composition of *B. atrophaeus* var.*globigii* and clearly separates the species by whole-genome phylogenetic analysis.

In this study, we generated a high-quality, closed reference genome for the 1942 isolate using a combination of 454, Illumina, and directed Sanger sequencing. We expect the final genome to have an error rate of ∼1 in 50,000 basepairs. When we mapped the 454 datasets for all of the isolates back to the finished sequence that was generated using the same DNA, we noted several putative SNPs that were common to all datasets (Table 4). We believe these represent errors introduced during generation of the final consensus sequence, as they did not appear when the isolates were mapped against draft sequence generated exclusively using the 454 platform; these are currently being verified and the final sequence will be updated.

Our sequences of multiple, closely related strains of this organism allow us to trace the derivation of the “military” BG isolates currently in use to a culture present at Camp Detrick during the 1940s and 1950s. The origin of ATCC 49822 is not as clear, but a publication from that era suggests a possible common origin at the University of Wisconsin [43]. While that strain is unlikely to be NRS-356 itself, given the presence of several strain-specific SNPs in our sequence, the SNPs common to both 49822 and the “military” lineage suggest a common ancestor that is not represented among the strains sequenced for this study. Given the lack of original records, it is unclear whether the NRS-356 variant in this study might have passed through Camp Detrick and been returned to the University of Wisconsin. However, given the date on the label and the general secrecy of operations at Camp Detrick during the Second World War [26] we consider this possibility unlikely.

During development of BGas a simulant for *B. anthracis*, strains were selected that exhibited the most desirable characteristics, those being rapid growth, high spore yield, and experimental reproducibility. Without being aware of the nature of the genetic alterations in their “optimized” strains, BW workers at Camp Detrick selected a mutant that provided dramatically higher total and relative spore yields, and generated consistent experimental results [6]. These strains were adopted into the inventories of numerous biodefense laboratories and have been used for many decades in simulations of decontamination and dispersal [12]. By applying a combination of genomic and biochemical profiling techniques, our data demonstrate that the BG isolates were “enhanced” by researchers at Camp Detrick during the development of the organism as a simulant.

The selection of a strain with the desired properties appears to have occurred in at least two discrete steps, as shown by the genome sequences and metabolic profiles. The initial step appears to have been the adaptation of a strain to growth in corn steep liquor, an acidic medium rich in protein and lactate [44]. The robust growth of the Detrick strains relative to 1942 in low-pH medium containing high lactate levels is likely due to mutations in *mmgD* (2-methylcitrate synthase, position 2029530), or a short-chain 3-oxoacyl-[acyl-carrier-protein] reductase (position 3437350), or both. The most likely candidate for a mutation in the Detrick isolates that increases growth is the frameshift in *mmgD*that occurred following the divergence from the 49822 lineage and results in an altered C-terminus (Figure S1). The *mmgD* geneencodes a 2-methylcitrate synthase that is expressed in the mother cell at the intermediate stages of sporulation [45]. A null mutation in *mmgD* had no perceptible effect on sporulation, although other TCA-cycle enzymes when mutated led to a loss of sporulation [45]. The effects of the frameshift mutation on sporulation and cellular physiology on the function of the enzyme are not clear at this time. We speculate that the frameshift mutation alters the substrate specificity of MmgD in favor of citrate, thus increasing the flux of lactate-derived intermediates through the tricarboxylic acid cycle. Evidence for this possibility includes the observations that 2-methylcitrate synthases can have partial citrate synthase activity [45] and that the *B. subtilis mmgD* gene can complement a *gltA* (citrate synthase) mutant of *E. coli* [46]. Alternatively, alteration of function of *mmgD* may have predisposed the lactate-adapted strain to acquisition of a hypersporulating phenotype, which is not readily isolated or stable in *B. subtilis *(see below); however the presence of a hypersporulating phenotype in an independently evolved lineage (1013) of BG indicates that the species may have an intrinsic predisposition to evolving such a phenotype *in vitro*.

The “military” strains also grow more readily on media containing *D,L*-diaminopimelic acid (*meso-DAP*), a major component of bacterial peptidoglycan. Corn steep liquor is derived from the incubation of corn in water at 42–55°C, during which a lactic fermentation by a community of wild organisms including numerous uncharacterized *Bacillus* spp. occurs. Total bacterial counts at the conclusion of CSL production can be quite high [44], thus the availability of such compounds for growth is not surprising. Another potential source of *meso*-DAP could be bacterial autolysis during sporulation. The relative roles of each of the alleles in growth on lactate and/or *meso*-DAP is the subject of current investigation in our laboratory.

**Figure 8:**
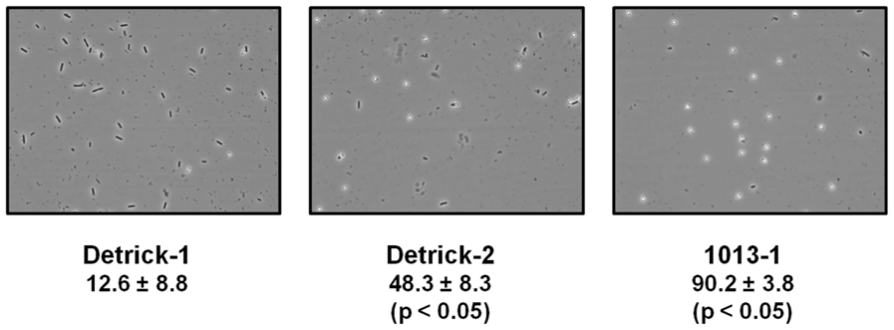
Multiple alignment of Spo0F protein sequences. The predicted protein sequences of Spo0F from multiple *Bacillus* species were aligned using ClustalW. Residues mutated in hypersporulating variants are indicated with grey (A98P) and black (H101R) arrows. Key: Batroph – *Bacillus atrophaeus*; Bsubtilis – *B. subtilis* 168; B_amyloliq – *B. amyloliquefaciens*; B_NRRL - *Bacillus*. spp. NRRL; B_SG-1 – *Bacillus* spp. SG-1; B_thur – *B. thuringiensis* strains Al Hakam and var. Israelensis (Isr); B_coahuil – *B. coahuilensis*; B_weihenst – *B. weihenstephanensis*; B_pseudomyc – *B. pseudomycoides*.

The second step in the development of BG as a simulant appears to have been the deliberate selection of a hypersporulating variant [6], [47]. Importantly, the selection of a strain optimized for spore yield resulted in the fixation of a new *spo0F* allele that has no counterpart among the available *spo0F* sequences (Figure 8). The sole Spo0Fsequence that differs at position 101 is that of *B. clausii*, in which tyrosine replaces histidine. Notably, the *spo0F(H101R)* mutation is distinct from a separate *spo0F(A98P)* mutation present in the *in vitro* passaged 1013 isolates. Given that the amino acid sequence of *B. atrophaeus* Spo0F is identical to that of *B. subtilis* but for two conservative substitutions, it is likely to have very similar if not identical biochemical properties. Detrick-1 and 1942 likely represent one of the two R colony morphotypes described by Hayward *et al.* [6], whereas the hypersporulating F morphotypes likely arose due to the emergence of the *spo0F(H101R)* mutation. However, the possibility that Detrick-1 represents a reversion mutant at this locus from Detrick-2 cannot formally be excluded, but since it represented the dominant morphotype in the 1952 Detrick vial we believe this is unlikely. The presence of the *spo0F(H101R)* allele in the ATCC 9372 strains suggests that these strains were acquired by ATCC after this mutation appeared within the Detrick lineage. Experiments to verify the roles of each allele in modulating sporulation are currently in progress. Preliminary results indicate that transformation of *B. subtilis* Δ*spo0F* with *B. atrophaeus* DNA and selection of *spo+* cells dramatically alters colony morphology independently of the *spo0F* allele introduced; additional studies to verify the effects of each allele are currently in progress (James Hoch, personal communication).

The H101R and A98P allelesare likely to alter the response to signals promoting sporulation. A*spo0F*(H101A) allele results in a sporulation-proficient strain that throws off sporulation-deficient papillae [48], and the same mutation has been shown to suppress the spo^−^ phenotype of a strain containing a defective *kinA* allele. H101 has been proposed as a potential metal-binding site with particular affinity for Cu^2+^ [49]. Binding of Cu^2+^ (or another divalent metal) at this site may modulate interaction with one or more sensor kinases that promote sporulation. Substitution of positively charged arginine at this position could potentially mimic the binding of a metal cation in the loop containing H101, resulting in altered sporulation of the strains due to a change in the interaction with the kinases governing sporulation. It is unclear why, given the proposed role of divalent Cu^2+^ in suppressing sporulation, H101R would result in a hypersporulation phenotype. The mechanistic relationship between *spo0F*(H101R) and the hypersporulation phenotype will be tested in future experiments.

Both variants in the 1013 lineage possess an A98P allele in *spo0F*. Although the presence of several other mutations within this lineage confounds the attribution of the hypersporulating phenotype to this allele at this time, the presence of a mutation in the same gene as another hypersporulating mutant is highly suggestive. The effect of proline substitution at position 98 on Spo0F functionis not immediately obvious, but the relatively inflexible proline residue can disrupt alpha-helices in protein structures. The 1013-1 lineage exhibits a hypersporulating phenotype even more pronounced than *spo0F(H101R)* strains in the “military” lineage. The observation that hypersporulating phenotypes have emerged during cultivationof two independent *B. atrophaeus* lineages point to the possibility that certain *in vitro* selection pressures may actually favor hypersporulating variants.

The selection pressures acting on the sporulation pathwayare highlighted by the sheer number of mutations discovered within the entire data set that occur in proteins known to play roles in sporulation. Nine of the 38 mutations (23%) found in all lineages were in genes that directly or indirectly regulate either entry into stationary phase or sporulation; this number exceeds the number that would be expected if mutations were to occur by chance, since less than 5% of *B. subtilis* genes are dedicated to regulatory processes of any kind [50], [51]. In addition to the mutations found within the “military” lineage, the two variants of ATCC 49822 shown in Figure 2 differ by mutations in *rpoB* (Table S5) which also plays a role in entry into sporulation [52]. Null mutations in *spo0F* resulting in asporogenous phenotypes contribute to colony morphology variation in *B. anthracis*, *B. thuringiensis* and *B. subtilis* [53], [54], [55]. Enhanced *in vitro* “fitness” is also a likely driver behind the recovery of asporogenic *B. anthracis* mutants that were discovered during the investigation into the *B. anthracis* attacks of 2001 [56]. Because the process of sporulation is highly energy-intensive and irreversible once commenced, mutants that delay sporulation (or fail to sporulate altogether) to take advantage of remaining nutrients would out-compete wild-type cells during repeated passage *in vitro* in the absence of other selection pressures, as has been demonstrated in extended *in vitro* evolution studies with *B. subtilis* under relaxed sporulation conditions [57]. This may not be universally the case, since gain-of-function mutations in sporulation such as those observed in this studymay compete favorably with wild-type cells if cannibalism of vegetative cells by sporulating bacteria is the dominant selective pressure [58]. Finally, horizontally transferred genetic elements can have dramatic effects on sporulation: for example, recent studies of phage lysogeny in *B. anthracis* have revealed the ability of several integrated phages to positively affect the kinetics of sporulation upon lysogeny of commonly used *B. anthracis* strains [59].

This study identifies the *spo0F(H101R)* allele as the signature of a deliberate selection during the development of *B. atrophaeus* as a simulant. However, without the knowledge of the history and the analysis of the phenotypes of the strains originating from “Camp Detrick” as published in the open literature, attribution of this genotype to a deliberate selection event would not have been definitive, since a similar phenotype is observed in the 1013 lineage which to our knowledge was not deliberately selected for any specific trait. Any study designed to determine genomic “signatures” of deliberate enhancement or selection is likely to require an analysis of the baseline likelihood that mutations conferring a similar phenotype would emerge and become fixed by natural processes within an evolutionary timeframe consistent with a known time interval or number of passages.

Available evidence suggests that hypersporulation is not easily evolved *in vitro*. Maughan and coworkers attempted to evolve populations of a laboratory strain of *B. subtilis* with a hypersporulating phenotype by repeatedly heat-shocking cultures. While their efforts to enrich for hypersporulators failed, other studies revealed that asporogenous mutants evolved readily [60], [61], confirming many early studies ([62] and references therein). With the exception of the studies by Maughan *et al.*, most ofthese investigators applied selections intended to inhibit sporulation rather than to enrich for strains with elevated sporulation rates. The 1013 lineage was never heat-shocked during its many transfers; thus the adaptations seen in this work are the result of balancing sporulation versus vegetative growth for prolonged periods on agar slants. However, because undomesticated isolates were observed to sporulate to 98–100% [60], we cannot formally exclude the possibility that *in vitro* culture of the 1942 strain following its isolation for an unknown period by the University of Wisconsin might have selected for a hyposporulating variant. In this scenario, the H101Rand A98P mutations would represent suppressor mutations. We consider this possibility unlikely, given the phenotypic similarity of two environmental isolates in the UW collection (1942 and NRS-356). Furthermore, a progression toward darker pigmentation and greater hemolysisis evident in the “military” lineage (Figure 2B). These phenotypic changes are associated with the accumulation of additional mutations including a P145L substitution mutation in *sigH*, a positive regulator of sporulation [63], [64] and an A13P mutation in *scoC*, a negative regulator of sporulation [65]. Together, the strains analyzed in this study suggest strong selective pressures on the genes in the sporulation pathway, and more carefully controlled studies should be carried out to determine the dynamics of *in vitro* evolution and adaptation of spore-forming organisms, as has been done extensively in *E. coli* [66], [67], [68], [69], [70].

Unexpectedly, the “military” lineages were also marked by the loss of catalase activity, whose presence is an identifying feature of both *B. subtilis* and *B. atrophaeus* [13]. This activity was present in a separate lineage of *in vitro* passaged organisms, so it is not immediately clear why “military” isolates, i.e. those subjected to selection within the early days of the development of BG as a simulant organism, would have lost the catalase activity characteristic of the parental isolate. Because the KatA gene product is not found in spores [41], [71], we consider it unlikely that the absence of this activity would impact the resistance of spores to decontamination reagents, and thus any antioxidant resistance phenotype exhibited by spores of “military” isolates would likely have gone unnoticed. However, direct comparisons of the “military” *B. atrophaeus* lineages to the progenitor strains have not been done, and pleiotropic effects of a *spo0F* mutation on spore physiology cannot currently be excluded.

Whole-genome approaches are becoming critical components of microbial forensics. The SNPs and indels identified in the analysis of evidentiary materials currently become the basis for higher-throughput assays to screen large numbers of samples [56], [72]. Decreasing costs of whole-genome sequencing, and the comprehensive nature of the analysis, may make this the preferred method of forensic analysis of microbial samples in the future. With recently developed techniques of allele quantitation within populations by mass spectrometry [73], real-time PCR [74], and census-by-sequencing [68], [75], it may be possible to quantitate accurately rare alleles within any given microbial population. We are particularly intrigued by the possibility that, given a mixture of different variants and sufficient sequencing power, ultra-high coverage sequencing may prove to be a more quantitative means of enumerating the relative populations in a sample even before the presence of variants has been established. The results from sequencing two strains of BACI051 in this study provide evidence of such hidden diversity.

The genomic basis of interlaboratory strain variation is only beginning to become evident, with recent studies tracing the histories of commonly used lab strains of *B. subtilis* 168, *E. coli*, *Salmonella enterica* serovar Typhimurium 14028s, *Pseudomonas aeruginosa*PA01 and *Mycobacterium tuberculosis* H37Rv [76], [77], [78], [79], [80], [81]. These have revealed significant divergence of putatively identical strains from one laboratory to another, largely arising from mutations that accumulate during serial passage. Like the earlier work, our study highlights the utility of approaches based on whole-genome sequencing for the discrimination of closely related strains, especially when investigating the provenance for a given isolate. Tragically, at least 13 institutions are known to have destroyed archival collections of Select Agents [82] following the implementation of mandatory monitoring and reporting requirements, representing an incalculable loss of phenotypic and genomic diversity. This report underscores the importance of maintaining the genetic heritage preserved in the culture collections of individual investigators and institutions.

## Supporting Information

Figure S1 Effect of frameshift mutation in the *mmgD* gene on the C-terminus of the 2-methylcitrate synthase homolog of *B. atrophaeus* strain Detrick-1. Arrow indicates the location of the GA dinucleotide insertion. Multiple alignment was performed using the ClustalW algorithm in the MEGAlign module of LaserGene.
(TIF)

Table S1 Table S1 is a consolidated spreadsheet containing Newbler HCDiffs calls for each templated assembly to the finished sequence of the 1942 isolate.
(XLSX)

Table S2 Table S2 details the scaffolding of large contigs of the *de novo* assembly of 454 data for the1942 strain based on optical maps.
(XLSX)

Table S3 Table S3 contains RAST annotation files (.gtf format) of the 1942 strain and indicates the location of the large deletions in ATCC 9372-1 and 1013-2.
(XLSX)

Table S4 Table S4 contains Omnilog phenotypic array data normalized to 1942 strain.
(XLSX)

Table S5 Table S5 contains HCDiffs calls from templated assembly of ATCC 49822 variants using the finished sequence of the 1942 isolate.
(XLSX)

Methods S1 Confirmation of BG Identity by RT-PCR.
(DOCX)
