# Supplementary figures and images for: Genomic Signatures of Strain Selection and Enhancement in Bacillus atrophaeus var. globigii, a Historical Biowarfare Simulant
Source: PLoS One. 2011 Mar 25;6(3):e17836. doi: 10.1371/journal.pone.0017836 (PMC3064580; doi:10.1371/journal.pone.0017836)

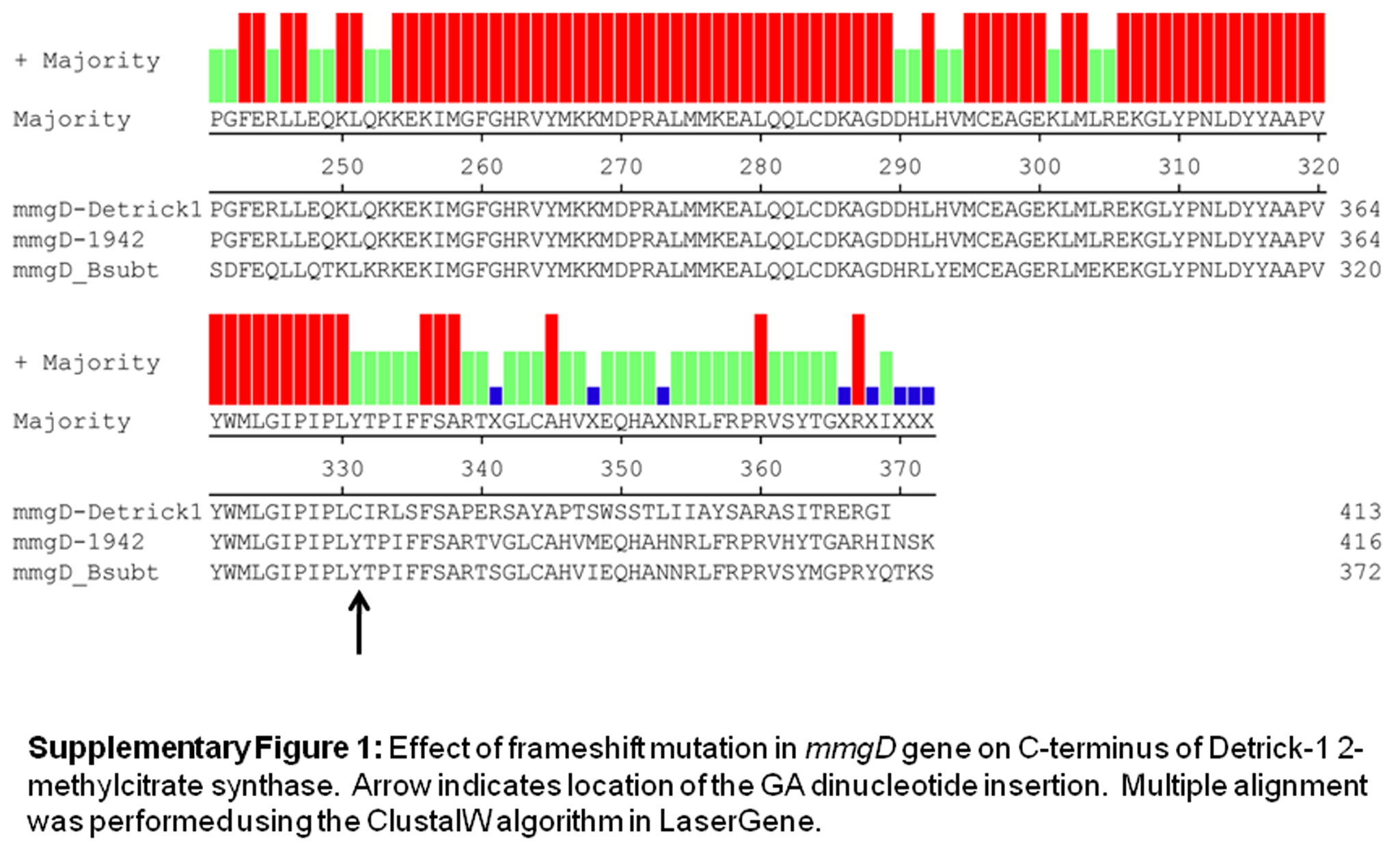

Supplement: Figure S1 — Effect of frameshift mutation in the mmgD gene on the C-terminus of the 2-methylcitrate synthase homolog of B. atrophaeus strain Detrick-1. Arrow indicates the location of the GA dinucleotide insertion. Multiple alignment was performed using the ClustalW algorithm in the MEGAlign module of LaserGene. (TIF) [file pone.0017836.s001.tif]
