# Supplementary material for: Genomic Signatures of Strain Selection and Enhancement in Bacillus atrophaeus var. globigii, a Historical Biowarfare Simulant
Source: PLoS One. 2011 Mar 25;6(3):e17836. doi: 10.1371/journal.pone.0017836 (PMC3064580; doi:10.1371/journal.pone.0017836)
Supplement: Methods S1 — Confirmation of BG Identity by RT-PCR. (DOCX) [file pone.0017836.s007.docx]

Supplementary Methods

Real Time PCR confirmation of *B. atrophaeus –* All isolates were confirmed to be *Bacillus atrophaeus* by polymerase chain reaction (PCR) analysis using the Cepheid SmartCycler^®^ (software version 1.2d). A 100 μl sample was removed from overnight liquid cultures in LB and placed into a 200 µl ABI MicroAmp tube. Genomic DNA was released from the cells by heating at 96°C for 3 minutes. Ten microliters of boiled material was transferred into a 100 µl SmartCycler^®^ reaction tube containing 90 µl PCR Master Mix. Each 100 µl reaction contained 1X 5mM MgCl_2_ PCR Buffer (Idaho Technologies, Cat: 1768), 200 mM dNTP mix (Invitrogen, Cat: 18427-088), 0.3 mM forward and reverse primer (Integrated DNA Technologies; BG_Forward: 5’-ACC AGA CAA TGC TCG ACG TT-3’; BG_Reverse: 5’-CCC TCT TGA AAT TCC CGA AT-3’), 0.2 mM FAM/TAMRA probe (Perkin Elmer, BG_Probe: 5’-FAM-ACT GAA CAG CTG ATC GAG ACA GCT GCA-TAMRA-3), and 4 units of Platinum^®^ Taq DNA Polymerase (Invitrogen, Cat: 10966-034). Primers targeted the *recF* gene of *B. atrophaeus* to produce a 132 bp amplicon. The PCR was carried out using the following cycling parameters: 95°C for 15 seconds followed by 50 cycles of 95°C for 1 second and 60°C for 6 seconds.
